# Global and regional prevalence and burden for premenstrual syndrome and premenstrual dysphoric disorder: A study protocol for systematic review and meta-analysis

**DOI:** 10.1097/MD.0000000000028528

**Published:** 2022-01-07

**Authors:** Mingzhou Gao, Hao Zhang, Zhan Gao, Xunshu Cheng, Ya Sun, Mingqi Qiao, Dongmei Gao

**Affiliations:** a Innovation Research Institute of Traditional Chinese Medicine, Shandong University of Traditional Chinese Medicine, Jinan, Shandong Province, China; b Experimental Center, Shandong University of Traditional Chinese Medicine, Jinan, Shandong Province, China; c College of Traditional Chinese Medicine, Shandong University of Traditional Chinese Medicine, Jinan, Shandong Province, China.

**Keywords:** burden, premenstrual dysphoric disorder, premenstrual syndrome, prevalence, systematic review

## Abstract

**Introduction:**

Premenstrual syndrome (PMS) and premenstrual dysphoric disorder (PMDD) are becoming common mental diseases in women impairing daily functioning. Estimation of the epidemiological burden of PMS/PMDD can serve as scientific basis for prevention and management of premenstrual disorders. Herein, we firstly provide a protocol to perform estimation on the prevalence and risk factors for PMS/PMDD in the general population globally and regionally.

**Methods/design:**

The PubMed, Web of Science, Chinese National Knowledge Infrastructure, the Cochrane Central Register of Controlled Trials (Cochrane Library), Chinese VIP Information, EMBASE, Wanfang Database, as well as the Chinese Biomedical Literature Database will be queried to find related studies containing information on the prevalence of PMDD (2011–2021). Two independent reviewers will comb the literature and abstract the data characteristics. Disparities will be reconciled via consents. The primary outcome will be the global prevalence. The random-effect model will be employed to pool the assessments. The standard *χ*^2^ tests, as well as the *I*^2^ statistic will be used to determine heterogeneity. Furthermore, the meta-regression analysis will be employed to estimate the differences in study-level characteristics. All the statistical analyses will be carried out in the software Stata v 15.0 (Stata Corporation, College Station, TX), as well as the R (v R 3.5.1, R Foundation for Statistical Computing, Vienna, Austria) software.

**Discussion:**

Based on existing evidence, our study will offer a high-quality synthesis for global and regional prevalence, burden, and risk factors of PMS/PMDD. Effective strategies will be made for prevention and management of epidemiological burden on the PMS/PMDD, even premenstrual disorders.

**Ethics and dissemination:**

This study does not involve the specific patients, and all research data comes from publicly available professional literature, so an ethics committee is not required to conduct an ethical review and approval of the study.

**INPLASY registration number:**

INPLASY2021120065.

## 1 Introduction

Premenstrual dysphoric disorder (PMDD) is an severe type of premenstrual syndrome (PMS), typified by cyclical mood alterations leading to clinically marked distress, as well as functional impairment in reproductive age women.^[1,2]^ Established studies showed that an estimated 90% of females of reproductive age were impacted by mild to acute premenstrual symptoms. Among them, about 20% to 40% encounter PMS, while 2% to 8% experience PMDD.^[3]^ Similarly, it appears that the PMDD prevalence differs depending on culture, as well as ethnic group.^[4]^ For example, the PMDD prevalence in a nationwide sample of Korean women is 2.4%,^[5]^ 3.3% in a Bulgarian population,^[4]^ 7.7% among female university students in Jordan,^[6]^ and even 17.6% in young adult women in southern Brazil.^[7]^ Herein, global and regional studies focusing on the prevalence are required for appropriate and more precise exploration of the global prevalence of PMS/PMDD.

Recent studies indicated that generalized anxiety disorder along with bipolar disorder often occur together with PMDD,^[8]^ and women with PMS/PMDD are a high risk group for suicidality.^[9]^ Furthermore, the increased illness burden is associated with comorbidity between PMDD and bipolar disorder.^[10]^ Disruption of parenting and partner relationships and decreased productivity in work roles have become main burden of illness of PMDD.^[11]^ And it is demonstrated here that PMS/PMDD is linked to considerable burden on both physical, as well as mental attributes of health-related quality of life.^[12,13]^

Thus, efficacious treatments are necessary to lessen the individual suffering, as well as the influence on family, society, and economy for PMS/PMDD.^[14]^ In addition, a systematic review and meta-analysis will be carried out to provide profound insights into the prevalence, as well as develop approaches to lessen the socioeconomic burden of PMS/PMDD globally.

## 2 Methods and analysis

### 2.1 Protocol and registration

The present review protocol has been registered within the INPLASY database and registration number is INPLASY2021120065 (https://inplasy.com/inplasy-2021-12-0065/). This review will be conducted as per the preferred reporting items for systematic review and meta-analysis protocols’ principles.^[15]^ Moreover, this systematic review will be reported as per the preferred reporting items for systematic review and meta-analysis 2009 principles.

### 2.2 Literature search

Eight databases comprising PubMed, Chinese National Knowledge Infrastructure, EMBASE, Chinese Biomedical Literature Database, Wanfang Database, the Cochrane Central Register of Controlled Trials (Cochrane Library), Web of Science, as well as Chinese VIP Information will be systematically queried—from database inception until April 15, 2022. The querying strategy will be modified based on PubMed by employing the Mesh subject headings blended with free-text terms referring to published papers.^[16,17]^

**Figure 1:**
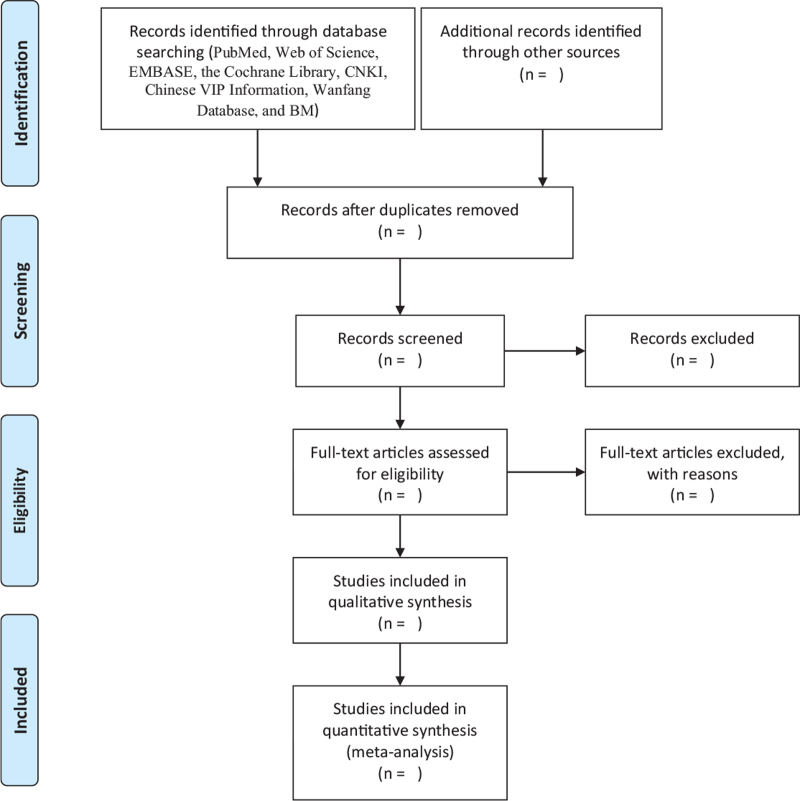
The PRISMA flow chart of the selection process.

Thus, search terms and syntax for PubMed will be: (“Premenstrual Syndrome” [Title/Abstract] OR premenstrual syndrome [MeSH Terms] OR premenstrual syndromes [MeSH Terms] OR premenstrual tension [MeSH Terms] OR syndrome, premenstrual [MeSH Terms] OR Premenstrual dysphoric disorder [Title/Abstract]) OR Disorder, Premenstrual Dysphoric [MeSH Terms] OR Dysphoric Disorder, Premenstrual [MeSH Terms] OR Premenstrual Dysphoric Syndrome [MeSH Terms] OR Syndrome, Premenstrual Dysphoric [MeSH Terms] AND (prevalence [Title/Abstract] OR prevalence [MeSH Terms] OR analysis, cross sectional [MeSH Terms] OR “Cross sectional” [Title/Abstract] OR Observational [Title/Abstract] OR “Case control” [Title/Abstract] OR Cohort [Title/Abstract] OR epidemiology [Title/Abstract] OR incidence [Title/Abstract]). Other databases will additionally be exhaustively queried for linked literature via the query terms applied in PubMed for each database correspondingly. Based on the inclusion, as well as the exclusion criteria, two reviewers will read the title, abstract, and entire text of the article for inclusion and exclusion screening. Dissensions between investigators will be reconciled via a consent and, if necessary, a third investigator will mediate and settle the differences. Studies will be entered in the Endnote, and eliminate duplicates via the Endnote function “remove duplicates.” The whole process of study screening and selection is shown in Figure 1.

### 2.3 Selection criteria

#### 2.3.1 Inclusion criteria

Included studies had to be population-based and reported the prevalence of PMS/PMDD or risk factors for PMS/PMDD, with any kind of random or nonrandom sampling in reproductive age females, in the common or school-hinged populace, across all ethnicities, educational backgrounds, socioeconomic, as well as living in all countries globally.

#### 2.3.2 Exclusion criteria

This study will factor out clinical or interventional articles, and studies focused on the PMS/PMDD prevalence with comorbidities, as well as researches involving high-risk populations or particular populace with specific ethnic background, educational, as well as socioeconomic backgrounds. In addition, we will exclude articles involving the validity, as well as reliability of the questionnaires.

### 2.4 Data collection and analyses

#### 2.4.1 Data extraction and management

EndNote 9.3.3 will be used to manage studies. Data of included studies will be collected from each eligible article and conducted independently in a standardized form by two authors. Discrepancies in the abstracted data will be settled via consent or mediation by the third author. The characteristics of the study will be recorded, including authors, publication year, journal, study populace, country, as well as city where the research was carried out, sample size, response rate, study design, race, gender, instruments, form of sampling, age, sample populace (based on general/school), diagnostic criteria, as well as measurement results.

#### 2.4.2 Quality assessment

The STROBE checklist will be utilized for quality evaluation of the enrolled cross-sectional researches.^[18,19]^ Through this evaluation strategy, we will explore six core constituents: study design, outcome data subjects, bias, descriptive, as well as measurement. Study quality of enrolled articles will be independently investigated by two investigators. Disagreements will be reconciled via consents, and third investigator will mediate and reconcile the case, if necessary.

#### 2.4.3 Statistical analyses and data synthesis

A meta-analysis on the prevalence of PMDD will be conducted using Stata (V.15.0. StataCorp, 2017), and employ the graphical approaches, as well as fixed or random effect models to combine the prevalence estimates. Statistical models will be employed in estimating trends via different research variables including gender, region, and age. Heterogeneity between the enrolled articles will be explored via the *I*^2^ heterogeneity statistic, and subgroup, as well as sensitivity analyses will be applied in assessing the heterogeneity sources. In addition, article bias will be examined via graphical approaches and statistical tests. If the articles will be markedly heterogeneous, as well as data pooling will not be possible, we will describe the results using tables, as well as figures.

#### 2.4.4 Publication bias

The Stata V.15.0 will be used for the analyses. Begg's test and Egger's test will be used to assess the publication bias of the included studies and form the publication bias plot.

## 3 Discussion

The purpose of this research is to yield a systematic review and meta-analysis protocol of global and regional prevalence and burden for PMS/PMDD. We contemplate that the data of this research will be employed by policy makers, as well as other stakeholders, and it will offer a path to further research at national, regional, as well as global levels.

## Acknowledgments

The authors would like to acknowledge the support of Team of Research and Innovation Focusing on Emotional Diseases and Syndromes in Shandong University of Traditional Chinese Medicine, Team of Young Scientific Research and Innovation Focusing on Pharmacology Mechanism of Emotional Diseases and Syndromes in Ganzangxiang, and the editors for reviewing the article.

## Author contributions

**Conceptualization:** Mingzhou Gao.

**Data curation:** Xunshu Cheng, Ya Sun.

**Formal analysis:** Ya Sun.

**Funding acquisition:** Mingqi Qiao, Dongmei Gao.

**Investigation:** Hao Zhang.

**Resources:** Zhan Gao.

**Software:** Mingzhou Gao, Hao Zhang, Zhan Gao, Xunshu Cheng.

**Supervision:** Mingqi Qiao, Dongmei Gao.

**Writing – original draft:** Mingzhou Gao.

**Writing – review & editing:** Mingzhou Gao.
